# Survival of anterior cruciate ligament  reconstruction in patients with Ehlers-Danlos syndrome: A comparison with anatomic risk factors in existing literature

**DOI:** 10.1007/s00264-025-06632-y

**Published:** 2025-08-07

**Authors:** Sebastian Schmidt, Chilan Bou Ghosson Leite, Alexander Bumberger, Domenico Franco, Cale Andrew Jacobs, Lars Richardson, Nikolaos Paschos, Simon Goertz, Eric Berkson, Peter Asnis, Christian Lattermann

**Affiliations:** 1 University Medical Centre Mannheim, University of Heidelberg, Mannheim, Germany; 2 Brigham and Women’s Hospital, Boston, MA USA; 3 University Hospital Vienna, Medical University of Vienna, Vienna, Austria; 4 Fondazione Policlinico Universitario Campus Bio-Medico, Rome, Italy; 5 Massachusetts General Hospital, Boston, United States

**Keywords:** ACL, Anterior cruciate ligament, Ehlers-Danlos syndrome, EDS

## Abstract

**Background:**

Ehlers-Danlos Syndrome (EDS) is a connective tissue disorder characterized by joint hypermobility, ligamentous laxity, and frequent joint injuries. These features could increase the risk of anterior cruciate ligament (ACL) tears, typically managed through ACL reconstruction (ACLR). Surgical intervention in EDS is challenging due to potential complications such as poor wound healing and tissue fragility. Limited evidence exists regarding the outcomes of ACLR in EDS patients; therefore, in this study we aimed to evaluate survival rates of primary and revision ACLR and examine associated demographic and anatomic risk factors for failure after ACLR.

**Methods:**

A retrospective review of 21 EDS patients (25 knees) who underwent ACLR between 1993 and 2023 was conducted. Patients with vascular EDS were excluded. Demographic and surgical data, including graft type, cause of injury, concomitant procedures such as meniscus repair and anatomical measurements (posterior tibial slope, intercondylar notch width, lateral femoral condyle morphology, tibiofemoral rotation and tibial tubercle- trochlear groove distance), were collected. Survival analysis was performed using Kaplan–Meier curves, with endpoints defined as ACLR failure or conversion to total knee arthroplasty (TKA). A multivariable survival analysis was used to identify predictors of outcomes. In addition, the influence of demographic and anatomical factors on the development of concomitant injuries and concomitant procedures were assessed. Anatomical factors were then compared with non-EDS patients from the existing literature.

**Results:**

The overall survival rate was 85.7% at mean follow-up of 50 months. Primary ACLR showed significantly higher survival rates 93.8% compared to revision ACLR 62.5% at 50 months (p = 0.03). Sports injuries, particularly skiing, were the leading cause of ACL tears (62%). Anatomical differences, such as increased lateral femoral condyle ratio (LFCR) and tibiofemoral rotation (TFR), were observed compared to non-EDS, ACL-intact patients from the literature (p < 0.01). However, these factors did not predict failure or influence concomitant injuries.

**Conclusion:**

This study demonstrates that ACLR in EDS patients achieves good survival rates. Anatomical risk factors differed significantly from non-EDS, ACL-intact patients, but were not predictive of failure, highlighting ligamentous laxity as the primary challenge.

## Introduction

Ehlers-Danlos syndrome (EDS) is a group of heritable, connective tissue disorders characterized by joint and skin laxity, and connective tissue fragility, often accompanied by chronic pain, increased distress and functional limitations, all resulting from abnormal collagen caused by different genetic mutations [1, 2] [2, 3]. While the journey to an accurate diagnosis is often prolonged due to the unclear aetiology of symptoms [1] and the absence of definitive genetic markers, the diagnosis of EDS currently relies on established clinical criteria [4]. These include generalized joint hypermobility, defined by a Beighton score of ≥ 5 out of 9 points, the presence systemic manifestations of connective tissue abnormalities (e.g., skin fragility, increased bruising and bleeding tendencies, vascular complications), a positive family history, musculoskeletal pain or joint instability, and the exclusion of other connective tissue disorders, such as rheumatologic diseases or skeletal dysplasia [4].

EDS has been estimated to impact 1 in 5,000 people worldwide, with the hypermobility type being the most prevalent [5]. Many individuals with this type experience hyperlaxity, which is frequently associated with joint subluxations or dislocations [3, 6]. This condition, combined with the excessive range of motion caused by hypermobility, may increases the risk of intra-articular injuries, including meniscal and ligament tears in the knee [7].

Tear of the anterior cruciate ligament (ACL) is among the most frequent knee injuries, particularly in young individuals engaged in sport activities involving cutting, pivoting, or kicking [8]. Surgical reconstruction is the standard treatment for active patients, aiming to restore knee stability and prevent secondary injuries [9, 10]. Overall, ACL reconstruction (ACLR) yields favorable long-term outcomes, with graft survival rates exceeding 85% at ten to 15 years postoperatively and high rates of return to sports [11–13]. However, outcomes can be influenced by several factors, including patient age, graft choice, concomitant injuries, and biomechanical alignment. In recent years, joint hypermobility has emerged as a significant risk factor for poorer outcomes after ACLR, including higher rates of graft elongation, persistent instability, and revision surgery [14–17]

For EDS patients, there are no standardized guidelines for addressing ACL injuries, and the evidence supporting the effectiveness of ACLR in this population remains limited [7, 14, 18]. Existing literature has largely focused on technical feasibility, with most reports consisting of isolated case studies and short-term follow-up [18]. Additionally, surgical management of EDS is particularly challenging due to increased risks of general surgical complications, such as intraoperative bleeding, delayed wound healing, and infections [18–20]. Therefore, the purpose of this study is to evaluate the demographic and knee anatomical parameters of patients with EDS sustaining an ACL injury and report on the ACLR survival in this population. Furthermore, known anatomical risk factors will be compared with the data of non-EDS patients from the literature and factors that may predict failure or concomitant injuries will be identified.

## Methods

The institutional review board approved the protocol for this study (protocol no. 2022P003141). A retrospective database search was conducted using the institution’s Research Patient Data Repository (RPDR), a clinical data registry that compiles medical record data from the healthcare system, to identify patients with hypermobile EDS. The RPDR was queried on 10/21/2024 for all patients diagnosed with hypermobile EDS (ICD-10 Q79.60) at the institution between 1993 and 2023. Diagnosis of EDS was confirmed by the Division of Human Genetics, as defined by the 2017 international diagnostic criteria [4].

Electronic medical records were reviewed to identify patients with ACL reconstruction. Those who underwent revision ACLR were also considered. Patients with follow-up of less than six months were excluded.

The indication for ACLR was ACL rupture with clinical signs of knee instability or failed conservative treatment. Demographic parameters, including age, sex, laterality of ACL injury, concomitant injuries, cause of injury and follow-up time, and intra-operative data regarding the type of graft and concomitant procedures, if any, were recorded. Additionally, any intra- or postoperative complications were noted. For subsequent analysis, patients were divided into two groups: primary ACLR (those with no signs of failure until the last follow-up) and revision ACLR. In addition, the influence of demographic and anatomical factors on the development of concomitant injuries was assessed. Furthermore, demographic and anatomical factors of the patients were compared based on the type of concomitant procedures performed, including chondroplasty, lateral extra-articular tenodesis (LET), and meniscectomy versus meniscus repair. All procedures were performed arthroscopically and with a medial portal drilling technique for the femoral tunnel.

While the postoperative treatment of all ACLR patients changed over the 30-year study period, all patients were managed according to the institution’s standardized postoperative rehabilitation protocol. This included protected weight bearing with crutches and a knee brace for the first few weeks, if tolerated. Likewise, all patients had in common that they were allowed to return to full activities after six months once a functional testing was performed.

Preoperative radiographs and magnetic resonance imaging (MRI) studies were identified, and the following parameters were assessed using previously described methods [21–25]: posterior tibial slope (PTS), intercondylar notch width (INW), lateral femoral condyle (LFC) bone morphology, tibia tubercle- trochlea groove (TT-TG) distance, tibiofemoral rotation (TFR). Briefly, PTS was defined as the angle between the tangent line to the tibial plateau and a line perpendicular to the tibial proximal anatomical axis on radiographic images using a plain true lateral view of the knee joint [26, 27].

The INW was measured on the coronal T2 MRI scans by identifying the coronal planes of the middle point of Blumensaat’s line. The full contours of the medial and lateral condyles, along with the notch morphology and the groove of the popliteus tendon sulcus on the lateral condyle, were visible on the scans. A line parallel to the epicondyles was drawn through the largest groove of the popliteus tendon sulcus. In relation to this line, the INW was measured [28].

The LFC bone morphology was evaluated on a radiographic plain true lateral view of the knee using the LFC Ratio [23, 29]. The long axis of the distal femur was identified by drawing a line between two circles, spaced 5 cm apart, centered on the femoral shaft, with the distal circle at the trochlea's most proximal point. The femoral condyle axis was established by drawing a line from the most posterior to the most anterior point of the lateral condyle. The distance from the intersection of these axes to the posterior point was then divided by the total anteroposterior length of the condyle and expressed as a percentage [23, 29].

The TT-TG distance was measured on axial T2 MRI scans by drawing a line perpendicular to the posterior femoral condylar axis that intersects the deepest point of the trochlear groove, along with a parallel line passing through the center of the patellar tendon insertion at the tibial tuberosity. The distance between these two lines in mm corresponds to the TT-TG distance [24, 30].

TFR was defined as the rotational alignment between the distal femur and proximal tibia using axial T2 MRI scans. The posterior femoral condylar axis was first identified at the level of the trochlea's deepest point. Subsequently, the posterior tibial condylar axis was determined at a level just distal to the tibial plateau and proximal to the fibular head [31]. The angle formed between these two axes was defined as the tibiofemoral rotation angle (TFA). Positive values indicated internal rotation of the proximal tibia relative to the distal femur [32].

### Statistical analysis

Continuous variables are reported as mean ± standard deviation (95% confidence interval; range). Normality was assessed using the Shapiro–Wilk test. Comparisons between groups for continuous variables were performed using the Student t-test or Mann–Whitney *U* test, when appropriate. Additionally, age and anatomic risk factors from this study were compared with measurements of non-EDS patients of other studies using the Kruskal–Wallis test, with Bonferroni correction applied to adjust for multiple comparisons. Categorical variables are described as absolute numbers with percentages, and comparisons between groups for categorical variables were performed using the Chi-square test or Fisher exact test where appropriate. Survival analysis was conducted using Kaplan–Meier curves with endpoints defined as ACLR failure (including graft rupture confirmed by clinical examination and imaging, or the need for revision ACL reconstruction due to functional instability) or conversion to total knee arthroplasty (TKA), and differences between primary ACLR and revision ACLR were assessed using the log-rank test. For competing risks analysis, cumulative incidence functions were estimated to account for competing events, and differences between groups were tested using Gray's test [33]. Cox proportional hazards models were used for multivariable survival analysis to identify predictors of outcomes. Model fit and performance were evaluated using the likelihood-ratio test, Wald test, and Score test. A significance threshold of P < 0.05 was applied for all tests. Statistical analyses were performed using R (Version 4.2.2).

## Results

A total of 21 patients (25 knees) with a confirmed diagnosis of hypermobile EDS who underwent ACL reconstruction were included, comprising 19 patients (19 knees) with primary ACLR and five patients (6 knees) with revision ACLR. Demographic analysis revealed significant differences between the primary ACLR group and the revision ACLR group in terms of age (27.3 ± 9.34 vs. 40.5 ± 12 years; p = 0.04) and follow-up (67.9 ± 56.3 vs. 28.8 ± 22.4 months; p = 0.03). All patients reported traumatic injury as the cause of their ACL tear, most commonly from sport activities (n = 13; 62%), with skiing being the most prevalent (n = 7; 33%). The most used graft type was bone patella tendon bone (BPTB) autograft (n = 9; 36%), followed by hamstring autograft (n = 4; 16%) and hamstring allograft (n = 4; 16%). No intra or post-operative complications were described. Three revision ACLR patients needed a reoperation due to medial meniscus tear and were treated with meniscectomy (p < 0.01). The demographic data are displayed in Table 1.

**Table 1:** Patient and surgical characteristics. Demographic and operative data for all included cases are presented, including type of ACL reconstruction (primary vs. revision), laterality, graft type (BPTB autograft, hamstring autograft/allograft), concomitant procedures, and follow-up duration. All surgeries were performed arthroscopically using a medial portal drilling technique. ACLR, anterior cruciate ligament reconstruction, n.s. non-significant; PF, patellofemoral compartement; LFC, lateral femoral condyle; MFC, medial femoral condyle; TP, tibia plateau

Characteristics	Primary ACLR Group	Revision ACLR Group	
	*n* (%)	*n* (%)	
Sex			
Male	4 (21%)	0	
Female	14 (74%)	6 (100%)	
Divers	1 (5%)	0	
Laterality			
Left	5 (26%)	3 (50%)	n.s
Right	14 (75%)	3 (50%)	n.s
Graft type			
Allograft	6 (32%)	4 (66%)	n.s
Autograft	13 (68%)	2 (33%)	n.s
Tunnel placement			
Anatomic	18 (95%)	4 (66%)	n.s
Concomitant injury			
Chondral defect	PF: 3(16%); LFC: 1 (5%); MFC: 1(5%); TP: 0	PF: 1 (17%); LFC: 0; MFC: 2 (33%); TP: 1 (17%)	n.s
Meniscus tear	medial: 1(5%) lateral: 4 (21%) both: 4 (21%)	medial: 4 (66%) lateral: 0 both: 1 (17%)	n.s
Concomitant procedure			
Meniscus repair	4 (21%)	2 (33%)	n.s
(Partial) Meniscectomy	5 (26%)	3 (50%)	n.s
Chondroplasty	PF: 3(16%); LFC: 1 (5%); MFC: 1(5%); TP: 0	PF: 1 (17%); LFC: 0; MFC: 2 (33%); TP: 1 (17%)	n.s
LET	1 (5%)	2 (33%)	n.s
	mean ± SD (95% CI; range)	mean ± SD (95% CI; range)	
Age (years)	27.3 ± 9.34 (22.8–31.8; 14–44)	40.5 ± 12 (27.9–53.1; 27–58)	*p* = 0.042
BMI (kg/m^2^)	26.9 ± 6.21 (23.9–29.9; 18.1–41.4)	29.9 ± 4.44 (25.3–34.6; 25.6–36)	n.s
Beighton Score	6.5 ± 1.56 (5.6–7.4; 5–8)	6.2 ± 0.84 (5.16–7.24; 5–7)	n.s
Genu recurvatum	12 (63%)	3 (50%)	n.s
Follow-up (months)	67.9 ± 56.3 (40.8–95; 6–174)	28.8 ± 22.4 (5.32–52.3; 8–62)	*p* = 0.028

### Survival

**Fig. 1:**
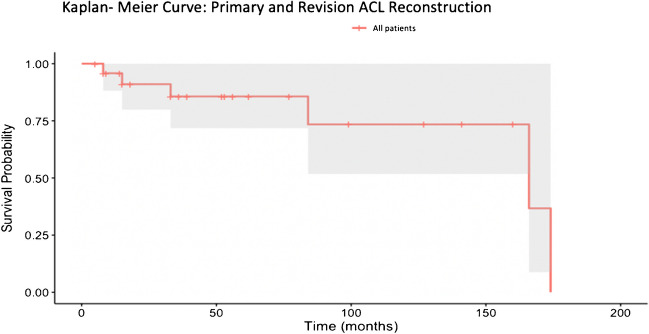
Kaplan- Meier curve of primary and revision anterior cruciate ligament reconstruction

The survival analysis demonstrated survival rates after 50 months of 85.7% (Fig. 1) with 13 patients at risk.

Patients in the primary ACLR group demonstrated higher survivorship over the follow-up period compared to those in the revision group, with survival rates after 50 months of 93.8% in the primary ACLR group and 62.5% in the revision ACLR group. After 150 months, the survival rate in the primary ACLR group remains at 78.1%. The number at risk decreases over time for both groups. At 50 months of follow-up, 11 patients remained at risk in the primary group and only one in the revision group. By 150 months, the number at risk dropped to three in the primary group and zero in the revision group.

**Fig. 2:**
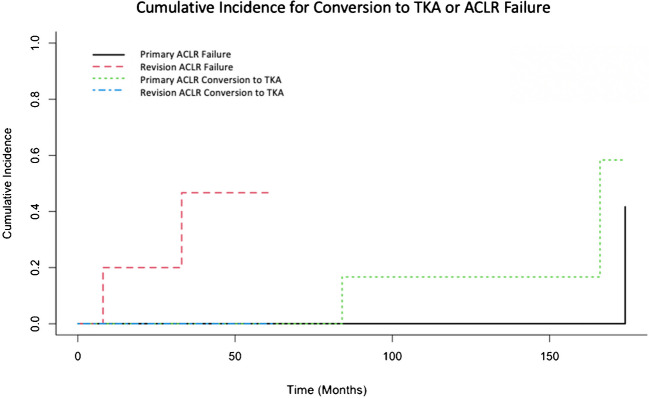
Cumulative incidence of the competing events conversion to TKA and ACLR failure. The risk of ACLR failure was significant higher in the revision group (*p* < 0.01)

The cumulative incidence of the competing events, conversion to TKA and ACLR failure, revealed significant differences in the risk of ACLR failure (p < 0.01) (Fig. 2).

The evaluation of the impact of clinical and demographic factors on the risk of failure showed no significant effects of the variables. After using a reduced model focusing on BMI and age, the analysis showed no significant association with adverse outcomes.

### Comparison of age and anatomic risk factors to non-EDS patients from the literature

#### Posterior tibial slope

The comparison with the patient cohorts (Group A: healthy controls; n = 1000; Group B: ACL-injured; n = 1000) of *Weiler *et al*.* [34] revealed significant differences between the age of EDS patients of this study and Group A (36.3 (range, 18–66); p < 0.01) as well as Group B (41.2 (range, 18–65); p < 0.01). The posterior tibial slope of the EDS patients was 9.28° ± 2.57°. There were no significant differences to the ACL-injured group (10.04° ± 3°) and none to the group of healthy ACL-intact controls (9.02° ± 2.9°).

#### Intercondylar notch width

Regarding the comparison between EDS patients and a healthy ACL-intact control group (n = 222; age, 33.3 ± 11.1) or an ACL-injury group (n = 308; age, 33.8 ± 12.5) in the study by *Fernandez *et al*. *[35], there was no significant difference in the age of the groups.

The comparison of INW between EDS patients (18.49 ± 1.77), healthy patients (19.5mm ± 3.6mm) and ACL-injured patients (18.2mm ± 3.1mm) was not statistically significant.

#### LFCR

Comparing the patient population of this study with that of *Pfeiffer *et al*. *[29](control group, n = 68, age, 29.8 ± 10.6; primary ACL-injury group, n = 50, age, 27.4 ± 9.4; failed ACLR group, n = 49, age, 25.7 ± 4.9), there are no significant differences for age. The EDS patients do not show significant differences regarding LFCR (66.2% ± 4.2%) to the patients with primary ACL-injury (64.2% ± 3.8%) or failed ACLR (64.4% ± 3.6%), but significant differences to the healthy control group (61.2% ± 2.4%; p < 0.01).

#### TFR

In terms of demographics, there were significant differences in age between the EDS patients and 182 healthy control patients in *Vassalou *et al. (41.8 ± 16.5; p < 0.01), whereas there were no differences in age between 151 patients with intact ACLR (25.9 ± 10) and 151 patients with failed ACLR (25.7 ± 10.4) in the study by *Leite *et al. [25, 30]. The EDS patients show significant differences regarding TFR (5.36° ± 3.66°) to the healthy control group (3.2° ± 3.3°; p = 0.03) and intact ACLR patients (3.0° ± 3.3°; p = 0.03) whereas there was no significant difference in the failed ACLR group (5.8° ± 4.5°).

#### TT-TG

The comparison of the TT-TG of the EDS patients (9.67mm ± 5.04mm) with the patient population of *Leite *et al*.* [30] showed significantly lower values compared to the intact ACLR cohort (13.2mm ± 3.7mm; p < 0.01) and the patients with failed ACLR (12.5mm ± 4.4mm; p < 0.01) (Table 2).

**Table 2:** Imaging measurements. LFCR, lateral femoral condyle ratio; TFR, tibiofemoral rotation; TT-TG, tibial tubercle-trochlear groove; n.s. non-significant; Tibial slope, posterior tibial slope

	mean ± SD (95% CI; range)	mean ± SD (95% CI; range)	mean ± SD (95% CI; range)	
Anatomic risk factors	Total	Primary ACLR Group	Rvision ACLR Group	
*Tibial Slope(°*)	9.28 ± 2.57 (8.27–10.29; 5–13)	9.30 ± 2.47(8.1–10.5; 5–13)	9.30 ± 3.14 (6–12.6; 5–13)	n.s
*Intercondylor wldth(mm)*	18.49 ± 1.77(17.77–19.21; 15.7–22.4)	18.6 ± 1.86(17.7–19.5; 15.7–22.4)	18 ± 1.48(16.5–19.6; 16.6–20.1)	n.s
*LFCR (%)*	66.2 ± 4.2 (64.5–67.8; 58–75)	65.7 ± 3.7(63.9–67.5; 58–71)	67.7 ± 5.8 (61.6–73.7; 58–75)	n.s
*TFR(°)*	5.36 ± 3.66 (3.83–6.89; 2–14)	5.76 ± 3.98 (3.87–7.66;−2–14)	4 ± 2 (2.25–5.75; 2–7)	n.s
*TT-TG(mm)*	9.67 ± 5.04 (7.56–11.78;·3–18.6}	9.94 ± 4.38 (7.64–12.23; 3–18.6)	8.75 ± 6.23 (3.32–14.24; 3–17.8)	n.s

#### Concomitant injuries

The demographic data and anatomical risk factors had no significant influence on the development of concomitant injuries (meniscus and chondral injuries). In a subgroup analyses between the meniscus repair group and the meniscectomy group a significant difference with regard to BMI (p = 0.025) was found. The differences for the chondroplasty and LET group were not significant.

## Discussion

The most important finding of this study is that primary ACLR in EDS patients leads to good midterm survival rates and acceptable short term survival rates in patients with revision ACLR. Survival probabilities for primary ACLR were significantly higher compared to revision ACLR in patients with EDS. These survival rates are similar to those reported in patients with generalized joint hyperlaxity (GJH) but without hEDS [36–38]. However, GJH patients have been shown to carry a higher risk of graft rerupture compared to individuals without hyperlaxity, and clinical outcomes may also be inferior [36, 37]. Whether these findings apply similarly to patients with hEDS remains unclear and should be further investigated in future studies. Regarding anatomic risk factors, only the lateral femoral condyle ratio (LFCR) and tibiofemoral rotation (TFR) showed significant differences compared to non-EDS and intact ACL controls (p < 0.01). These findings suggest that, despite the distinct connective tissue characteristics of EDS patients, successful ACLR is achievable, especially in primary reconstructions**.**

The results align with the broader orthopedic literature, which shows that revision ACLR patients generally have a higher risk of re-injury and lower patient-reported outcome measures (PROMs) compared to primary ACLR patients [15–17, 39]. However, this study uniquely establishes that EDS patients undergoing primary ACLR can achieve comparable survivorship to non-EDS populations [40, 41].

With regard to possible risk factors for ACLR failure, no factors were identified in this study. However, the literature highlights numerous risk factors in non-EDS populations such as age, sex, training regimen, early return to sport and biomechanical deficits [15, 16]. The use of allografts has also been associated with higher ACL graft rerupture rates [17]. This was also not observed in this current study.

Another factor that is emerging as a key contributor to ACLR failure is insufficiently restored rotational stability. Numerous studies have shown that the use of lateral extra-articular tenodesis leads to a significant reduction in the rerupture rate, particularly in hypermobile patients [15, 42, 43]. Only a small number of patients in our cohort underwent LET, and accordingly, no statistically significant differences in outcomes were observed. However, the low frequency of LET procedures limits the ability to draw definitive conclusions.

In addition, several osseous morphologic characteristics have been associated with an increased risk of ACL injury or failed ACLR [29]. One of the most investigated factors is the posterior tibial slope [21, 26, 27, 29, 34]. A PTS of > 12° has been established as a risk factor for failure after ACLR [34, 44]. This association was not be demonstrated in this study, suggesting that the hypermobility in these patients is primarily due to soft tissue imbalance rather than bone-related factors.

Awareness of the bone morphology of the distal femur is increasing in recent research [23, 29]. Specifically, the morphological and morphometric characteristics of the LFC, such as an increased posterior femoral condylar depth, are thought to play a significant role in the occurrence of ACL injuries [23, 29]. This may be due to an increased posterior depth of the LFC, which leads the femur to a more oval shape. This alteration can cause greater anisometry and elongation of the lateral and anterolateral knee structures, potentially reducing the contact surface between the femur and tibia [29]. As a result, there may be an increase in rotational knee laxity, particularly near full extension [29]. The data from this current study suggests that this relationship may also exist in patients with EDS.

While factors such as internal tibial rotation and TT-TG distance are routinely considered in the evaluation of patellofemoral instability, they are not commonly assessed in the context of ACL injuries [25, 30, 45]. However, radiographic measurements of axial rotation may be relevant to reinjuries following ACLR, especially in a patient population that is massively affected by patellofemoral instability [7, 18, 20, 30]. There is evidence that the TT-TG distance can be an additional tool for detecting increased rotational laxity after ACL injury, especially in combination with ALL injury [30, 45]. This finding does not appear to be relevant in our patient population, as the values were lower than in the study by *Leite *et al*.* and also lower than the threshold for healthy non-EDS patients [30, 45].

Regarding the TFR measured by the TFA, patients with intact ACLR presented similar TFAs to healthy intact controls [25, 30]. Our study found that the TFA was significantly higher in EDS patients compared to healthy controls and ACLR patients, underlining that the proposed threshold of 4.9° or greater is a sign for the diagnosis of ACL tears [25].

*Leite *et al*.* suggests that patients with ACLR failure exhibit greater internal tibial rotation compared to those with intact ACLRs [30]. Increased tibiofemoral rotation can place more reliance on secondary stabilizers, potentially leading to their earlier failure. This excess laxity may contribute to a higher risk of ACLR failure. In patients with EDS, these challenges are magnified due to the connective tissue abnormalities. To address this, adjunctive stabilizing procedures, such as anterolateral ligament (ALL) reconstruction or lateral extra-articular tenodesis, have been proposed to provide better rotational control of the knee [42, 43, 46].

The availability of outcome data for this patient group in the literature is very poor. A recent review looked at the outcomes after ACLR in EDS patients. In total, there were only six case reports with a maximum follow-up of 36 months, all of them reporting good outcomes, including no further knee instability and return to normal activity. These results are in line with the findings of our study. After successful ACLR, the patients showed no increased instability at the follow-up examinations.

Several studies have raised concerns about using autograft tissue in patients with EDS due to intrinsic changes in peri-articular connective tissues that compromise strength and stiffness [18, 46, 47]. Therefore, the use of allografts and lateral extra-articular tenodesis has been recommended for this patient population [42, 43]. However, in our cohort, both graft types were used and no significant differences in outcomes could be detected, likely due to the small sample size. However, in our cohort, both graft types were used and no significant differences in outcomes could be detected, likely due to the small sample size.

Currently, no evidence-based postoperative protocols exist that are specifically tailored to patients with EDS undergoing ACL reconstruction. All patients in our study followed the institutional standard rehabilitation regimen. However, given the characteristic delayed wound healing, joint instability, and risk of graft elongation in this population, protocol modifications, such as extended bracing duration, delayed initiation of sport-specific drills, or prolonged restriction from high-impact activities, may be warranted.

Importantly, this study contributes to the limited evidence available on ACLR in EDS patients. The data challenges traditional assumptions that connective tissue fragility precludes successful surgical intervention, providing a foundation for further research into graft selection and adjunctive procedures tailored to the unique needs of this population.

### Limitations

This study has several limitations. Its retrospective design and reliance on electronic medical records may have introduced selection bias and incomplete data. In addition, a minimum follow-up period of only six months was used, which may not be sufficient to capture long-term failures or complications. The small sample size (25 knees) reflects the rarity of EDS but substantially limits the statistical power to detect significant associations, especially in survival analysis and multivariable modeling. Therefore, nonsignificant findings in this study should be interpreted with caution, as the lack of statistical significance does not necessarily imply the absence of an effect. Furthermore, the study spans a period of 30 years (1993–2023), during which surgical techniques, graft choices, fixation methods, and rehabilitation protocols have evolved considerably. This temporal variability could have influenced outcomes by introducing heterogeneity in surgical management and postoperative care. For example, changes in graft preference from bone–patellar tendon–bone to hamstring autografts, advances in fixation devices, or shifts towards accelerated rehabilitation protocols may have affected graft survival and complication rates. However, to address this, we reported the smallest common denominators across all procedures, showing that despite differences in details, all surgeries shared the same base technique (arthroscopic reconstruction with medial portal femoral tunnel drilling) and a standardized rehabilitation protocol involving initial brace-protected weight bearing and return to full activities after functional testing at approximately six months. Due to the limited sample size, further subgroup analyses to adjust for temporal factors were not feasible. Additionally, the study lacks a matched control group for direct comparison of outcomes, but published results of ACLR in the general population allow comparison with existing literature as in other studies of this specific patient population [48].

To our knowledge, this is the first study to investigate the survival of ACLR and examine anatomical risk factors for ACLR failure in a cohort of patients with EDS. This research provides valuable insights into graft selection, risk factors, and injury mechanisms, particularly in rare populations such as those with EDS.

## Conclusion

This study demonstrates that ACLR in EDS patients achieves good survival rates. Anatomical risk factors differed significantly from non-EDS, ACL-intact controls, but were not predictive of failure, highlighting ligamentous laxity as the primary challenge.

## Data Availability

No datasets were generated or analysed during the current study.

## References

[1] Feldman ECH, Homan KJ, Williams SE et al (2023) A narrative review of the literature on illness uncertainty in hypermobile Ehlers-Danlos syndrome: implications for research and clinical practice. Pediatr Rheumatol Online J 21:121. 10.1186/S12969-023-00908-6 37845704 10.1186/s12969-023-00908-6 PMC10577933

[2] Gensemer C, Burks R, Kautz S et al (2020) Hypermobile Ehlers-danlos syndromes: complex phenotypes, challenging diagnoses, and poorly understood causes. Dev Dyn 250:318. 10.1002/DVDY.220 32629534 10.1002/dvdy.220 PMC7785693

[3] Petrucci T, Barclay SJ, Gensemer C et al (2024) Phenotypic clusters and multimorbidity in hypermobile Ehlers-Danlos syndrome. Mayo Clinic Proceedings: Innovations, Quality & Outcomes 8:253. 10.1016/J.MAYOCPIQO.2024.04.001 10.1016/j.mayocpiqo.2024.04.001 PMC11109295 38779137

[4] Malfait F, Francomano C, Byers P et al (2017) The 2017 international classification of the Ehlers-Danlos syndromes. Am J Med Genet C Semin Med Genet 175:8–26. 10.1002/AJMG.C.31552 28306229 10.1002/ajmg.c.31552

[5] Steinmann B, Royce PM, Superti-Furga A (2002) The Ehlers-Danlos syndrome. Connective tissue and its heritable disorders. pp 431–523. 10.1002/0471221929.CH9

[6] Grahame R (2008) Hypermobility: an important but often neglected area within rheumatology. Nat Clin Pract Rheumatol 4:522–524. 10.1038/NCPRHEUM0907 18762785 10.1038/ncprheum0907

[7] Ericson WB, Wolman R (2017) Orthopaedic management of the Ehlers-Danlos syndromes. Am J Med Genet C Semin Med Genet 175:188–194. 10.1002/AJMG.C.31551 28192621 10.1002/ajmg.c.31551

[8] Collins JE, Katz JN, Donnell-Fink LA et al (2013) Cumulative incidence of ACL reconstruction after ACL injury in adults: role of age, sex, and race. Am J Sports Med 41:544–549. 10.1177/0363546512472042 23302260 10.1177/0363546512472042 PMC3896975

[9] Sanders TL, Pareek A, Kremers HM et al (2017) Long-term follow-up of isolated ACL tears treated without ligament reconstruction. Knee Surg Sports Traumatol Arthrosc 25:493–500. 10.1007/s00167-016-4172-4 27221641 10.1007/s00167-016-4172-4

[10] Sanders TL, MaraditKremers H, Bryan AJ et al (2016) Incidence of and factors associated with the decision to undergo anterior cruciate ligament reconstruction 1 to 10 years after injury. Am J Sports Med 44:1558–1564. 10.1177/0363546516630751 26928338 10.1177/0363546516630751

[11] Arnold MP, Calcei JG, Vogel N et al (2021) ACL study group survey reveals the evolution of anterior cruciate ligament reconstruction graft choice over the past three decades. Knee Surg Sports Traumatol Arthrosc 29:3871–3876. 10.1007/s00167-021-06443-9 33486558 10.1007/s00167-021-06443-9

[12] Csapo R, Pointner H, Hoser C et al (2020) Physical fitness after anterior cruciate ligament reconstruction: influence of graft, age, and sex. Sports 8:30 32155933 10.3390/sports8030030 PMC7183074

[13] Barenius B, Ponzer S, Shalabi A et al (2014) Increased risk of osteoarthritis after anterior cruciate ligament reconstruction. 42:1049–1057. 10.1177/0363546514526139 10.1177/0363546514526139 24644301

[14] Brockmeyer M (2019) Editorial commentary: ligamentous hyperlaxity: a decisive factor for the indication of a combined anterior cruciate ligament and anterolateral ligament reconstruction? Arthroscopy 35:2655–2657. 10.1016/J.ARTHRO.2019.04.027 31500752 10.1016/j.arthro.2019.04.027

[15] Pache S, Del Castillo J, Moatshe G, Laprade RF (2020) Anterior cruciate ligament reconstruction failure and revision surgery: current concepts. Journal of ISAKOS 5:351–358. 10.1136/JISAKOS-2020-000457

[16] Belozo FL, Belozo RSMN, Ricardo Lopes C et al (2024) Anterior cruciate ligament: a brief narrative review of main risk factors for injury and re-injury. J Bodyw Mov Ther 38:92–99. 10.1016/J.JBMT.2024.01.022 38763622 10.1016/j.jbmt.2024.01.022

[17] Liechti DJ, Chahla J, Dean CS et al (2016) Outcomes and risk factors of rerevision anterior cruciate ligament reconstruction: a systematic review. Arthroscopy 32(10):2151–2159. 10.1016/J.ARTHRO.2016.04.017 27289278 10.1016/j.arthro.2016.04.017

[18] Schubart JR, Mills SE, Rodeo SA, Francomano CA (2024) Outcomes of orthopaedic surgery in Ehlers-Danlos syndromes: a scoping review. BMC Musculoskelet Disord 25:846. 10.1186/S12891-024-07937-6 39448975 10.1186/s12891-024-07937-6 PMC11515420

[19] Tinkle B, Castori M, Berglund B et al (2017) Hypermobile Ehlers-danlos syndrome (a.k.a. Ehlers-danlos syndrome type III and Ehlers-danlos syndrome hypermobility type): clinical description and natural history. Am J Med Genet C Semin Med Genet 175:48–69. 10.1002/AJMG.C.31538 28145611 10.1002/ajmg.c.31538

[20] Yonko EA, LoTurco HM, Carter EM, Raggio CL (2021) Orthopedic considerations and surgical outcomes in Ehlers-Danlos syndromes. Am J Med Genet C Semin Med Genet 187:458–465. 10.1002/AJMG.C.31958 34845816 10.1002/ajmg.c.31958

[21] Smith HC, Vacek P, Johnson RJ et al (2012) Risk factors for anterior cruciate ligament injury: a review of the literature — part 1: neuromuscular and anatomic risk. Sports Health 4:69. 10.1177/1941738111428281 23016072 10.1177/1941738111428281 PMC3435896

[22] Kiapour AM, Yang DS, Badger GJ et al (2019) Anatomical features of the tibial plateau predict outcomes of ACL reconstruction within 7 years after surgery. Am J Sports Med 47:303. 10.1177/0363546518823556 30640519 10.1177/0363546518823556 PMC6382545

[23] Ubl ST, Harmes JC, Koch E et al (2024) No clinically relevant relationship between different quantitative measurement methods of the lateral femoral condyle morphology on lateral radiographs in anterior cruciate ligament-injured patients. J Exp Orthop. 10.1002/JEO2.70078 39502323 10.1002/jeo2.70078 PMC11534868

[24] Schoettle PB, Zanetti M, Seifert B et al (2006) The tibial tuberosity-trochlear groove distance; a comparative study between CT and MRI scanning. Knee 13:26–31. 10.1016/J.KNEE.2005.06.003 16023858 10.1016/j.knee.2005.06.003

[25] Vassalou EE, Klontzas ME, Kouvidis GK et al (2016) Rotational knee laxity in anterior cruciate ligament deficiency: an additional secondary sign on MRI. Am J Roentgenol 206(1):151–154. 10.2214/AJR.15.14816 26700347 10.2214/AJR.15.14816

[26] Utzschneider S, Goettinger M, Weber P et al (2011) Development and validation of a new method for the radiologic measurement of the tibial slope. Knee Surg Sports Traumatol Arthrosc 19:1643–1648. 10.1007/S00167-011-1414-3 21298254 10.1007/s00167-011-1414-3

[27] Tensho K, Kumaki D, Yoshida K et al (2023) Does posterior tibial slope laterality exist? A matched cohort study between ACL-injured and non-injured knees. J Exp Orthop 10:1–8. 10.1186/S40634-023-00702-Z/TABLES/7 38057689 10.1186/s40634-023-00702-z PMC10700254

[28] Ficek K, Rajca J, Cholewiński J et al (2021) Analysis of intercondylar notch size and shape in patients with cyclops syndrome after anterior cruciate ligament reconstruction. J Orthop Surg Res. 10.1186/s13018-021-02706-w 34496898 10.1186/s13018-021-02706-w PMC8425156

[29] Pfeiffer TR, Burnham JM, Hughes JD et al (2018) An increased lateral femoral condyle ratio is a risk factor for anterior cruciate ligament injury. J Bone Joint Surg Am 100:857–864. 10.2106/JBJS.17.01011 29762281 10.2106/JBJS.17.01011

[30] Leite CBG, Merkely G, Farina EM et al (2023) Effect of tibiofemoral rotation angle on graft failure after anterior cruciate ligament reconstruction. Am J Sports Med 51:2291–2299. 10.1177/03635465231163856 37454271 10.1177/03635465231163856

[31] Thakrar RR, Al-Obaedi O, Theivendran K, Snow M (2019) Assessment of lower limb rotational profile and its correlation with the tibial tuberosity-trochlea groove distance: a radiological study. J Orthop Surg (Hong Kong). 10.1177/2309499019868148 31451047 10.1177/2309499019868148

[32] Diederichs G, Köhlitz T, Kornaropoulos E et al (2013) Magnetic resonance imaging analysis of rotational alignment in patients with patellar dislocations. Am J Sports Med 41:51–57. 10.1177/0363546512464691 23136177 10.1177/0363546512464691

[33] Dignam JJ, Kocherginsky MN (2008) Choice and interpretation of statistical tests used when competing risks are present. J Clin Oncol 26:4027. 10.1200/JCO.2007.12.9866 18711194 10.1200/JCO.2007.12.9866 PMC2654314

[34] Weiler A, Berndt R, Wagner M et al (2023) Tibial slope on conventional lateral radiographs in anterior cruciate ligament-injured and intact knees: mean value and outliers. Am J Sports Med 51:2285–2290. 10.1177/03635465231178292 37306059 10.1177/03635465231178292 PMC10353028

[35] Fernndez-Ja ÉNT, López-Alcorocho JM, Rodriguez-Iñigo E et al (2015) The importance of the intercondylar notch in anterior cruciate ligament tears. Orthop J Sports Med 3:2325967115597882. 10.1177/2325967115597882 26535388 10.1177/2325967115597882 PMC4622305

[36] Kim SJ, Choi CH, Lee SK et al (2018) Minimum two-year follow-up of anterior cruciate ligament reconstruction in patients with generalized joint laxity. J Bone Joint Surg Am 100:278–287. 10.2106/JBJS.17.00767 29462031 10.2106/JBJS.17.00767

[37] Larson CM, Bedi A, Dietrich ME et al (2017) Generalized hypermobility, knee hyperextension, and outcomes after anterior cruciate ligament reconstruction: prospective, case-control study with mean 6 years follow-up. Arthroscopy: The Journal of Arthroscopic & Related Surgery 33:1852–1858. 10.1016/j.arthro.2017.04.012 28599980 10.1016/j.arthro.2017.04.012

[38] Zsidai B, Piussi R, Thomeé R et al (2023) Generalised joint hypermobility leads to increased odds of sustaining a second ACL injury within 12 months of return to sport after ACL reconstruction. Br J Sports Med 57:972. 10.1136/BJSPORTS-2022-106183 37192830 10.1136/bjsports-2022-106183 PMC10423474

[39] Wright R, Spindler K, Huston L et al (2011) Revision ACL reconstruction outcomes - MOON cohort. J Knee Surg 24:289. 10.1055/S-0031-1292650 22303759 10.1055/s-0031-1292650 PMC4451059

[40] Rahardja R, Zhu M, Love H et al (2020) Rates of revision and surgeon-reported graft rupture following ACL reconstruction: early results from the New Zealand ACL Registry. Knee Surg Sports Traumatol Arthrosc 28:2194–2202. 10.1007/S00167-019-05773-Z 31679071 10.1007/s00167-019-05773-z

[41] Samuelsen BT, Webster KE, Johnson NR et al (2017) Hamstring autograft versus patellar tendon autograft for ACL reconstruction: is there a difference in graft failure rate? A meta-analysis of 47,613 patients. Clin Orthop Relat Res 475:2459. 10.1007/S11999-017-5278-9 28205075 10.1007/s11999-017-5278-9 PMC5599382

[42] Helito CP, Sobrado MF, Giglio PN et al (2019) Combined reconstruction of the anterolateral ligament in patients with anterior cruciate ligament injury and ligamentous hyperlaxity leads to better clinical stability and a lower failure rate than isolated anterior cruciate ligament reconstruction. Arthroscopy 35:2648–2654. 10.1016/J.ARTHRO.2019.03.059 31421960 10.1016/j.arthro.2019.03.059

[43] Sundemo D, Senorski EH, Samuelsson K (2021) Editorial commentary: diagnosis and treatment of generalized joint hypermobility in patients with anterior cruciate ligament injury. Arthroscopy: The Journal of Arthroscopic & Related Surgery 37:2348–2350. 10.1016/J.ARTHRO.2021.03.052 34226016 10.1016/j.arthro.2021.03.052

[44] Salmon LJ, Heath E, Akrawi H et al (2018) 20-year outcomes of anterior cruciate ligament reconstruction with hamstring tendon autograft: the catastrophic effect of age and posterior tibial slope. Am J Sports Med 46:531–543. 10.1177/0363546517741497 29244525 10.1177/0363546517741497

[45] Polat AE, Polat B, Gürpınar T et al (2020) Tibial tubercle-trochlear groove (TT-TG) distance is a reliable measurement of increased rotational laxity in the knee with an anterior cruciate ligament injury. Knee 27:1601–1607. 10.1016/J.KNEE.2020.08.014 33010779 10.1016/j.knee.2020.08.014

[46] Hishimura R, Kondo E, Matsuoka M et al (2022) Double-bundle anterior cruciate ligament reconstruction using autologous hamstring tendon hybrid grafts in a patient with hypermobile Ehlers-Danlos syndrome: a case report. Knee 35:81–86. 10.1016/J.KNEE.2022.02.005 35220136 10.1016/j.knee.2022.02.005

[47] Rombaut L, Malfait F, De Wandele I et al (2012) Muscle-tendon tissue properties in the hypermobility type of Ehlers-Danlos syndrome. Arthritis Care Res (Hoboken) 64:766–772. 10.1002/ACR.21592 22232076 10.1002/acr.21592

[48] Parikh SN, Nemunaitis J, Wall EJ et al (2024) Midterm outcomes of isolated medial patellofemoral ligament reconstruction for patellar instability in Ehlers-Danlos syndrome. Orthop J Sports Med 12(6):23259671241241096. 10.1177/23259671241241096 38845609 10.1177/23259671241241096 PMC11155334

